# Accelerator-Based Production of Scandium Radioisotopes for Applications in Prostate Cancer: Toward Building a Pipeline for Rapid Development of Novel Theranostics

**DOI:** 10.3390/molecules28166041

**Published:** 2023-08-13

**Authors:** Jason P. Meier, Hannah J. Zhang, Richard Freifelder, Mohammed Bhuiyan, Phillip Selman, Megan Mendez, Pavithra H. A. Kankanamalage, Thomas Brossard, Antonino Pusateri, Hsiu-Ming Tsai, Lara Leoni, Sagada Penano, Kaustab Ghosh, Brittany A. Broder, Erica Markiewicz, Amy Renne, Walter Stadler, Ralph Weichselbaum, Jerry Nolen, Chien-Min Kao, Satish K. Chitneni, David A. Rotsch, Russell Z. Szmulewitz, Chin-Tu Chen

**Affiliations:** 1 Department of Radiology, The University of Chicago, Chicago, IL 60637, USA; jasonmeier@uchicago.edu (J.P.M.); hjzhang@uchicago.edu (H.J.Z.); freifeld@uchicago.edu (R.F.); mohammedb@uchicago.edu (M.B.); npusateri@uchicago.edu (A.P.); penano@uchicago.edu (S.P.); kghosh@uchicago.edu (K.G.); brittany.broder@nist.gov (B.A.B.); arenne@uchicago.edu (A.R.); ckao95@uchicago.edu (C.-M.K.); schitneni@uchicago.edu (S.K.C.); 2 Integrated Small Animal Imaging Research Resource, Office of Shared Research Facilities, The University of Chicago, Chicago, IL 60637, USA; hmtsai@uchicago.edu (H.-M.T.); leoni@uchicago.edu (L.L.); ejmark@uchicago.edu (E.M.); 3 Cyclotron Facility, The University of Chicago, Chicago, IL 60637, USA; 4 UChicago/Argonne Joint Radioisotope Initiative (JRI), Chicago, IL 60637, USA; wstadler@uchicagomedicine.org (W.S.); rweichselbaum@uchicagomedicine.org (R.W.); nolen@anl.gov (J.N.); 5 Department of Medicine, The University of Chicago, Chicago, IL 60637, USA; pselman@bsd.uchicago.edu (P.S.); megan.mendez@medicine.bsd.uchicago.edu (M.M.); 6 Physics Division, Argonne National Laboratory, Lemont, IL 60439, USA; phettiach@bnl.gov (P.H.A.K.); brossard@anl.gov (T.B.); 7 Collider Accelerator Department, Brookhaven National Laboratory, Upton, NY 11973, USA; 8 National Institute of Standards and Technology, Gaithersburg, MD 20899, USA; 9 Department of Radiation and Cellular Oncology, The University of Chicago, Chicago, IL 60637, USA; 10 Medical Isotope Development Group, Oak Ridge National Laboratory, Oak Ridge, TN 37830, USA

**Keywords:** theranostics, PSMA-617, prostate cancer, mCRPC, PET, SPECT, TRT, Scandium-43, Scandium-47, radioisotopes

## Abstract

In the field of nuclear medicine, the β^+^ -emitting ^43^Sc and β^−^ -emitting ^47^Sc are promising candidates in cancer diagnosis and targeted radionuclide therapy (TRT) due to their favorable decay schema and shared pharmacokinetics as a true theranostic pair. Additionally, scandium is a group-3 transition metal (like ^177^Lu) and exhibits affinity for DOTA-based chelators, which have been studied in depth, making the barrier to implementation lower for ^43/47^Sc than for other proposed true theranostics. Before ^43/47^Sc can see widespread pre-clinical evaluation, however, an accessible production methodology must be established and each isotope’s radiolabeling and animal imaging capabilities studied with a widely utilized tracer. As such, a simple means of converting an 18 MeV biomedical cyclotron to support solid targets and produce ^43^Sc via the ^42^Ca(d,n)^43^Sc reaction has been devised, exhibiting reasonable yields. The ^Nat^Ti(γ,p)^47^Sc reaction is also investigated along with the successful implementation of chemical separation and purification methods for ^43/47^Sc. The conjugation of ^43/47^Sc with PSMA-617 at specific activities of up to 8.94 MBq/nmol and the subsequent imaging of LNCaP-ENZaR tumor xenografts in mouse models with both ^43/47^Sc-PSMA-617 are also presented.

## 1. Introduction

In nuclear medicine, the field of theranostics is predicated upon the use of theranostic pairs with a combination of complementary diagnostic and therapeutic capabilities that can be used in tandem. Within a theranostic pair, the diagnostic radiopharmaceutical allows for imaging and an accurate assessment of the state of the disease under study, while the therapeutic radiopharmaceutical delivers the desirable radiation to treat the disease effectively. When conjugated to a tumor-targeting ligand, these radionuclide pairs have demonstrated high efficacy in identifying, staging, and treating patients with high-risk and advanced tumors such as in metastatic castration-resistant prostate cancer (mCRPC) [1,2,3,4]. The greater the similarity between these radionuclides regarding chemical, biological, and pharmacokinetic properties in vivo, the greater the correlation between the acquired image and the delivered radiotherapeutic dose, providing physicians a more accurate representation of treatment progression [1,2,3,4].

Presently, ^68^Ga (t_1/2_ = 67.71(9) min) and ^177^Lu (t_1/2_ = 6.647(4) days) are clinically the most commonly used theranostic pair [5,6,7,8]. Other radionuclides frequently evaluated include the pairs of ^131^I/^123^I and ^99m^Tc/^90^Y [1,9].

^43^Sc (t_1/2_ = 3.891(12) h) decays by β^+^ emission with an 88.1% branching ratio, and the remaining decays occur through electron capture. Conversely, ^47^Sc (t_1/2_ = 3.3492(6) days) decays entirely through β^−^ emissions. Together, the complementary β^+^ and β^−^ emissions of ^43^Sc and ^47^Sc enable them to serve as a “true” theranostic pair for cancer therapy. The scandium true theranostic pair has several distinct advantages over traditionally used theranostic pairs, such as ^68^Ga and ^177^Lu: (1) As both radioisotopes are of the same element, they benefit from identical radiolabeling and chemical processes, making any production methods developed applicable to both nuclides; (2) radiopharmaceutical agents with the same element have identical binding characteristics to the target and in vivo pharmacokinetics, whereas tumor-targeting compounds labeled with nuclides of two different elements may have differential biological, chemical, and metabolic properties. As such, ^43^Sc positron emission tomography (PET) imaging provides improved fidelity of the magnitude and location of dosage, reflecting more accurately its radiotherapeutic counterpart, ^47^Sc, while PET images from one positron emitter, such as ^68^Ga, may not be completely representative of the localization of a β^−^ emitter of a different element such as ^177^Lu; (3) through the use of longer-lived isotopes, the applications of nuclear medicine can be further broadened through the expansion of the PET imaging window [10,11]. ^43^Sc’s half-life allows for extended imaging of the physiological and biological effects of this theranostic pair as well as higher-quality images at later time points relative to ^68^Ga [12]; (4) ^47^Sc emits a 159 keV γ-ray that is near-optimal for single photon emission computed tomography (SPECT) imaging [13,14,15]. While ^44^Sc has been more commonly investigated and shown promise in the context of Sc-based theranostics, it has a distinct disadvantage in the emission of a high-energy γ-ray (1157 keV, 100%) [13,15,16,17,18]. This γ-ray has the potential to down-scatter into the PET energy window, diminishing the quality of its images and depositing non-localized doses. In contrast, ^43^Sc provides a better quality of PET imaging and reduced toxicity to adjacent healthy tissue without high energy γ-ray emissions [19,20].

The Argonne National Laboratory has previously conducted preliminary investigations into the production of ^47^Sc using their Low-Energy Accelerator Facility (LEAF) through the ^48^Ti(γ,p)^47^Sc reaction using ^nat^TiO_2_ targets, achieving typical yields between 111 and 185 MBq and > 1110 GBq/mg [21]. At the University of Chicago Cyclotron Facility, bombardment of enriched ^42^CaCO_3_ targets by the IBA Cyclone 18/9 system’s 9-MeV deuteron beam produced ^43^Sc through the ^42^Ca(d,n)^43^Sc reaction, providing the foundation for future routine production of ^43^Sc at other medical cyclotron facilities.

Small-molecule prostate-specific membrane antigen (PSMA) inhibitor, PSMA-617, in conjunction with various radionuclides, namely ^68^Ga and ^177^Lu, has shown great promise in the diagnosis and treatment of mCRPC with PSMA overexpression, making labeled ^43/47^Sc an ideal candidate for preclinical studies. These studies will lay the foundation for further studies into potential radioscandium conjugation with other TRT ligands [5,6,7,8,17].

This work aims to establish a method to produce ^43^Sc and ^47^Sc, label it with PSMA-617 as ^43^Sc/^47^-Sc-PSMA-617, and investigate its utilization in a preclinical PSMA-positive prostate cancer model via PET and SPECT imaging studies. The results of this proof-of-concept study, a precursor to the full-fledged development of scandium theranostics, are herein described.

## 2. Results and Discussion

### 2.1. Cyclotron-Based Production of ^43^Sc

#### 2.1.1. Beam-Stop as a Target Holder

The synthesis of ^43^Sc can be accomplished through various production routes, with the ^42^Ca(d,n)^43^Sc reaction being favorable due to its high yields at beam energies less than 10 MeV and the relative affordability of ^42^Ca compared to the ^43^Ca utilized in the ^43^Ca(p,n)^43^Sc reaction [14,22,23,24]. Moreover, most cyclotrons geared towards PET-isotope production are offered with the deuteron beam capability, making it a feasible production route for many medical facilities.

**Figure 1:**
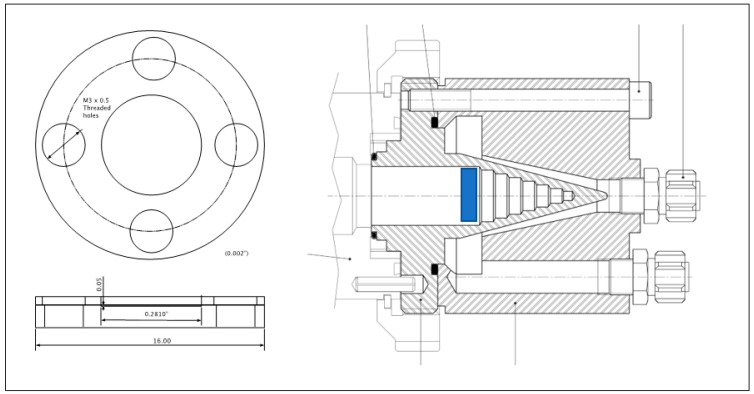
Schematic drawing of the target holder showing the backing disk and the ring that is joined to the backing to hold the ^42^Ca powder. The holder’s location within the beam stop is marked in blue, also exemplifying that the target-holder insert is loaded from the front of the beam stop (i.e., where the beam enters the beam stop when inserted) and cooling is provided by the back of the beam stop along the edges of the stepped cone.

**Figure 2:**
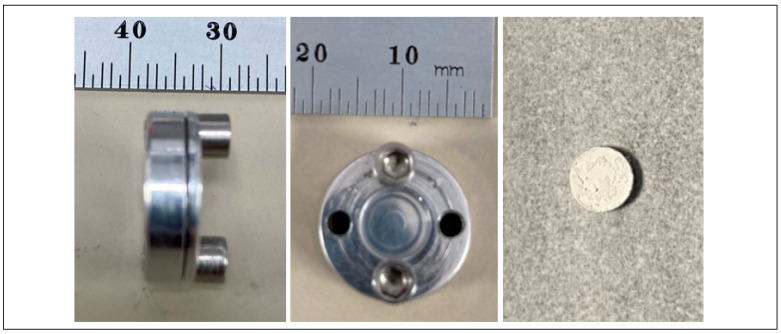
Photographs of the target holder and a representative ^42^CaO target.

In this study, an IBA Cyclone 18/9 cyclotron with deuteron capabilities was utilized. While the cyclotron lacks a solid target station, it is outfitted with two beam stops: water-cooled, aluminum, conically shaped stops capable of withstanding the maximum current of the accelerator. To produce the nuclide, one of the beam stops was converted into a solid target irradiation station through the design and fabrication of a solid target holder insert, consisting of a backing aluminum disk (5 mm thickness) with a thin aluminum ring (1.5 mm thickness) held against the disk with screws. The insert had a diameter of 16 mm and was designed to fit snugly into the beam stop to ensure proper cooling (see Figure 1 and Figure 2).

To form the target, ^42^Ca powder was pressed and sandwiched between two graphite foils, which were held against the aluminum backing disk with the ring before insertion into the beam stop. A small amount of diffusion pump oil is also applied to the sides and outer edge of the target holder to increase the heat transfer from the target-holder to the water-cooled beam stop. 

#### 2.1.2. Target Materials and Preparation

^42^CaO was prepared through the calcination of 94.37–96.30% isotopically enriched ^42^CaCO3 at 900 °C. The isotopic composition of the calcium material, along with potential impurities due to irradiation, is provided in Table 1. The resulting ^42^CaO material was pressed into a cylindrical pellet with a diameter of approximately 7 mm. Target masses were adjusted based upon the expected amount of activity necessary for subsequent experiments and ranged from 4.6–22.4 mg. Targets were stored in a container wrapped in parafilm to minimize CO_2_ reabsorption.

Once the pellet had been prepared, the backing aluminum disk of the target holder was placed onto a jig that holds all pieces in place. On top of the disk, a first graphite foil was placed. Then, the ^42^Ca pellet was carefully slid onto the graphite foil and a second foil was placed over it. Finally, the retaining aluminum ring was set on top of the outermost foil and the M3 screws were threaded into the backing disk to hold the entire assembly together. The excess graphite was trimmed off with a razor blade, the diffusion pump oil was applied to the target body, and the target was inserted into the beam-stop, as shown in Figure 1.

#### 2.1.3. Target Irradiation

The target was irradiated with a 9 MeV deuteron beam for a duration of approximately 8 h. Over several iterations, a variety of beam currents were used, ranging from 1–8 µA. After bombardment, the beam-stop/target-assembly was allowed to cool for one hour before the beam-stop was removed.

The target holder was manually removed from the beam-stop. The dose rates of the beam-stop were between 1–2 R/h (10–20 mGy/h) on contact. Typical body doses were 10–25 mR after removal of the target holder and disassembly. The graphite/calcium/graphite layers of the target are easily and quickly separated from the target holder and placed in a PTFE beaker. A summary of target masses and resulting yields are shown in Table 2. 

**Figure 3:**
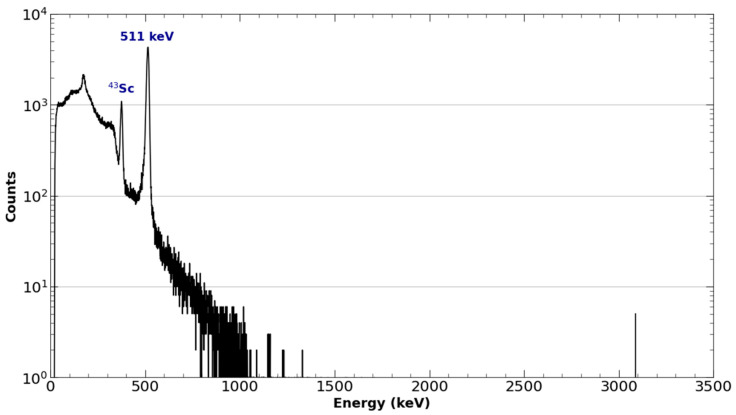
A logarithmic γ-spectrum of ^43^Sc taken from the solid target and graphite foils acquired one hour after the end of bombardment at 30.48 cm using a RayMon 10 handheld CZT detector.

To verify the purity of ^43^Sc activity obtained from the irradiation process, a Raymon10 cadmium zinc telluride (CZT) detector was used to obtain a gamma spectrum of a 962.0 MBq-irradiated target at 12 inches shortly after being removed from the target holder (Figure 3). The resulting spectrum clearly shows the expected 511 keV peak from positron annihilation and ^43^Sc’s characteristic 372.8 keV gamma ray with no detection of contamination. No change of the spectrum’s characteristic shape was observed with any downstream purification or separation chemistry.

#### 2.1.4. Comparison to Theoretical Yield Calculations

Theoretical end-of-bombardment (EOB) yields of ^43^Sc from the ^42^Ca(d,n)^43^Sc reaction were calculated through the numerical solving of Equation (1), where *N_A_* is Avogadro’s number, *I* is the incident particle flux, *A_T_* is the atomic weight, *λ* is the decay constant, *t* is the beam time, *E* is particle energy, *E*_0_ is the incident particle energy, *E_e_* is the exit particle energy, *σ*(*E*) is the cross-section, and *S*(*E*) is the mass stopping power [25].

(1) $$Yield=\frac{N_{A}I}{A_{T}}(1-e^{-\lambda t})\int_{E_{e}}^{E_{0}}\sigma (E)\frac{dE}{S(E)}$$

**Figure 4:**
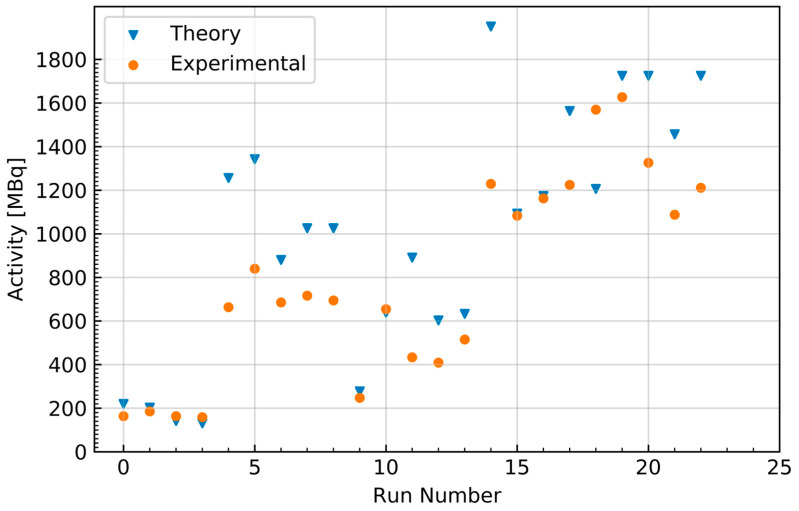
Activity at the end of bombardment for each ^43^Sc production run along with the theoretically estimated activity from the numerical solving of Equation (1).

**Figure 5:**
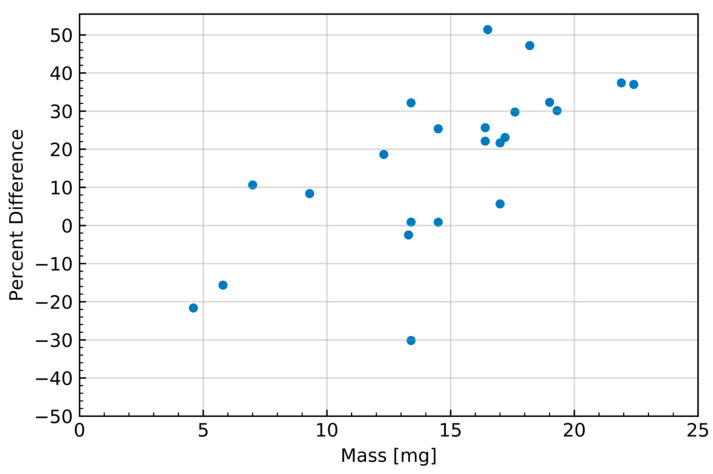
The percent difference of the experimentally obtained ^43^Sc yields relative to the numerically estimated yields as a function of target mass.

Cross-sections were obtained from TENDL simulations in the EXFOR database, as experimentally determined cross-sections are not presently defined for this energy range [26]. Mass stopping power was derived from simulations performed in ATIMA, as deuteron stopping power is not presently defined in calcium or calcium oxide. Theoretical EOB activity yields were compared to experimental yields with an average difference of 20.7%, as shown in Figure 4 and Figure 5. Most theoretical yields were shown to be overestimates of the experimentally obtained activity. This overestimate of the yield of the ^42^Ca(d,n)^43^Sc reaction is consistent with the current literature, which shows TENDL to significantly overestimate the reaction’s cross-section in the presently measured deuteron energy range (~3–7 MeV) [23].

Deviation of the experimental yield from the theoretical yield was also seen to generally increase with target mass, as shown in Figure 5; however, this correlation may also be the result of the theoretical yield’s dependence on the simulated stopping power in the numerical calculation rather than originating from the experiment results. Combined with the aforementioned discrepancies in cross-section in the literature, this suggests that there is a possibility that the observed differences in theoretical and experimental yield are the consequence of a lack of developed characterization of the ^42^Ca(d,n)^43^Sc cross-section and the interactions of deuterons in calcium oxide. The discrepancy between experimental and theoretical yields may also be the result of the culmination of a multitude of unevaluated uncertainties, such as well-counter accuracy and activity loss originating from the makeshift target holder. The current workflow requirements imposed by the daily routine cyclotron operation did not allow for more detailed measurements of these potential activity losses associated with the non-traditional use of the beam-stop space to accommodate the target holder.

#### 2.1.5. Separation and Purification

All reagents used in the separation, purification, and radiolabeling processes were trace analysis grade.

Once removed from the target holder and placed in a PFA beaker, dissolution began with the addition of 2 mL of 15.7 M HNO_3_. The resultant mixture was placed on a hotplate at 70 °C and with a magnetic stirrer at 180 RPM. While stirring, 4 mL of trace analysis grade water was added slowly over 30 min, followed by an additional 5 min of stirring.

The dissolved target solution was transferred to a 15 mL Falcon tube and centrifuged at 4000 RPM for 5 min. After centrifugation, the resulting supernatant was extracted with a syringe and loaded onto a preconditioned DGA cartridge (2 mL) for separation [12,14,21]. The column was then washed with 3 full column volumes (FCV) of 5 M HNO_3_ and 3 FCV of 1 M HCl. 95 °C 0.1 M HCl was used to elute trapped ^43^Sc from the DGA cartridge into ten 500 µL fractions as ^43^Sc-Cl_3_. Aliquots were assayed, and those of the highest concentration were used for radiolabeling.

### 2.2. Linear Accelerator-Based Production of ^47^Sc

#### 2.2.1. Target Preparation

**Figure 6:**
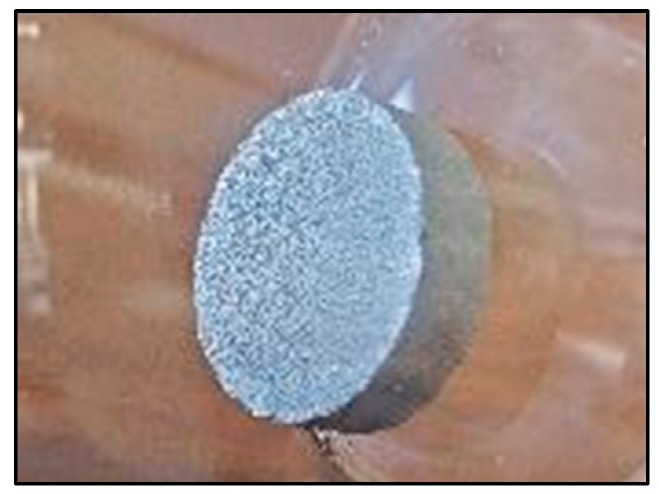
A representative natural titanium target utilized in the production of ^47^Sc.

Titanium metal targets (Figure 6) were prepared by pressing Ti powder (2.227 g/cm^3^_,_ 49% of theoretical density) with a hydraulic press by applying 1.5 ton of pressure for 5 min at ambient temperature in a 12.7 mm diameter die. Three pellets with mass of ~1 g were produced.

#### 2.2.2. Irradiation

The three Ti targets were stacked in a water-cooled aluminum target carrier and irradiated at the Low Energy Accelerator Facility (LEAF) at the Argonne National Laboratory. 

A 40 MeV electron beam at a current of 12.5 µA for 10–12 h irradiated six tantalum plates to produce Bremsstrahlung photons for the ^Nat^Ti(γ,p)^47^Sc reaction. The target carrier was placed behind the convertor so that the target was 20 mm from the last converter plate. The targets were allowed to cool for 10–16 h post irradiation and were then transported to a hood for processing. 

**Figure 7:**
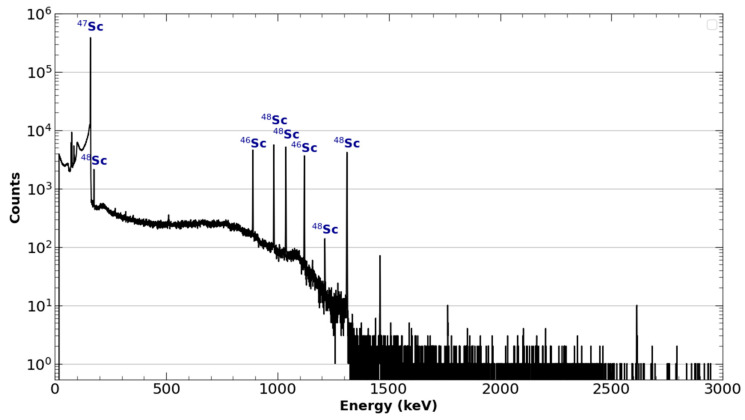
Logarithmically scaled γ-spectrum data of ^47^Sc taken from the solid target and foil taken after elution from the DGA column with HCl.

Since natural Ti was used as the target material, ^46^Sc, ^47^Sc, and ^48^Sc were observed in the products. The ratios observed were typically in the range of 0.7–1% ^46^Sc, 89–91% ^47^Sc, and 7–9% ^48^Sc decay corrected to EOB. These impurities are observed in the product’s gamma spectrum in Figure 7. The average EOB yield was approximately 925 MBq. Impurities can be reduced and the ^47^Sc maximized by using enriched ^48^Ti targets: for a 100%-pure ^48^Ti target, the only radionuclidic impurity would be from ^46^Sc produced via ^48^Ti(γ,pn)^46^Sc.

#### 2.2.3. Chemical Separation

The irradiated Ti targets were dissolved in 60 mL of concentrated HCl under reflux. After complete dissolution, indicated by the solution turning a deep purple color, the solution was allowed to cool for 30 min, then transferred to a graduated flask and diluted to 100 mL using washings from the Erlenmeyer flask. Similar to ^43^Sc separation, the separation of radioscandiums from the Ti target was completed using DGA resin. A DGA resin was prepared by soaking it overnight in 2.5 M HNO_3_ and decanting the fine particles. The resin was loaded into a plastic column, compressed with a sintered glass frit, and washed with 10 mL of water, followed by 3 M HCl, then 6 M HCl.

The target solution was gravity-fed to the DGA resin and washed with 6M HNO_3_ and 3M HCl. A final wash of 1 M HCl was used to lower the pH of the solution and enhance the elution of the product. Over 90% of radioscandiums were eluted with 15 mL of warm 0.1 M HCl. An oversized DGA column bed (0.75 g) was chosen based on the significant mass of Ti (3 g) from which Sc was to be separated. Smaller resin beds in similar separations have eluted the Sc product in smaller fractions [14]. Optimizations of this process have not been investigated.

The product solution was evaporated to dryness on a hot plate. The purity and molar activity were analyzed by inductively coupled plasma–mass spectroscopy and by complexation with DOTA. The product was typically void of common impurities (Cu, Fe, and Pb) below the method detection limit (MDL) [19]: typically, < 5 µg of total Ti was observed, and the Sc content was below the MDL value. Using the MDL value, the specific activity was typically > 25.9 TBq/mg. A typical molar activity of > 6 MBq/nmol DOTA was observed by complexation of the ^47^Sc with DOTA. These values are consistent with high-purity radioscandium products observed in the literature [12,14,27].

### 2.3. Radiosynthesis

#### 2.3.1. ^43^Sc-PSMA-617

**Figure 8:**
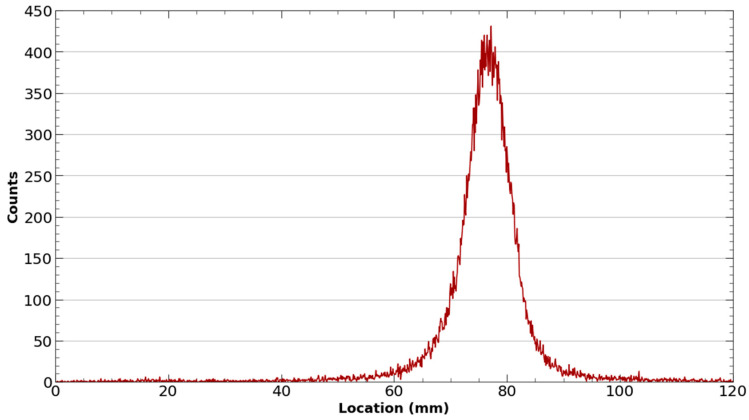
Representative iTLC of ^43^Sc-PSMA-617. The absence of a peak near the origin of the iTLC exhibits the successful radiolabeling of ^43^Sc-PSMA-617 with a yield of > 95%.

To better facilitate the conjugation of ^43^Sc to the DOTA chelator on PSMA-617 and minimize colloid formation, purified selected fractions of ^43^ScCl_3_ were adjusted to a pH between 4.2 and 5.0 through the addition of an equal volume of 0.5 M NH_4_OAc (pH 4.5) buffer. Once buffered, DOTA-PSMA-617 was added to the ^43^ScCl_3_/^43^Sc(OAc)_3_ solution and placed in a thermomixer at 95 °C and 500 RPM for 40 min. The radiolabeling reaction was monitored by spotting the reaction on an iTLC-SG paper developed on a 50:50 mixture of 1 M ammonium acetate and methanol and scanning with a Lablogic radio-TLC scanner (Figure 8).

**Figure 9:**
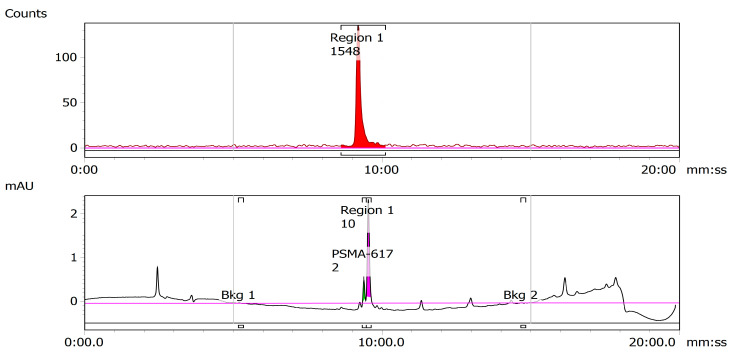
Representative HPLC chromatogram of ^43^Sc-PSMA-617. The proximity of the retention times of the UV trace of cold PSMA-617 (**bottom**) and the radioactive trace of the solutions of radiolabeled ^43^Sc-PSMA-617 and (**top**) indicate successful radiolabeling.

Once successful radiolabeling of >90% was confirmed, labeled activity was loaded onto a C-18 cartridge preconditioned with 6 mL of EtOH and 10 mL of water. The C-18 cartridge was then washed with 10 mL of water, and ^43^Sc-PSMA-617 was eluted with EtOH in 0.2 mL aliquots. Aliquots were then transferred to a 20 mL glass scintillation vial and placed in a rotary evaporator until EtOH was evaporated to near dryness. After evaporation, ^43^Sc-PSMA-617 was reconstituted in phosphate buffer solution (PBS), pH 7.4. The final molar activity of each ^43^Sc-PSMA-617 dose was calculated using a standard calibration curve generated from the UV trace of the reference, cold Sc-PSMA-617, and found to be 7.14 MBq/nmol, although successful labeling was performed up to 8.77 MBq/nmol, with gradual improvement seen each experiment. A representative HPLC chromatogram of the radiolabeled compound and cold trace can be seen in Figure 9.

#### 2.3.2. ^47^Sc-PSMA-617

**Figure 10:**
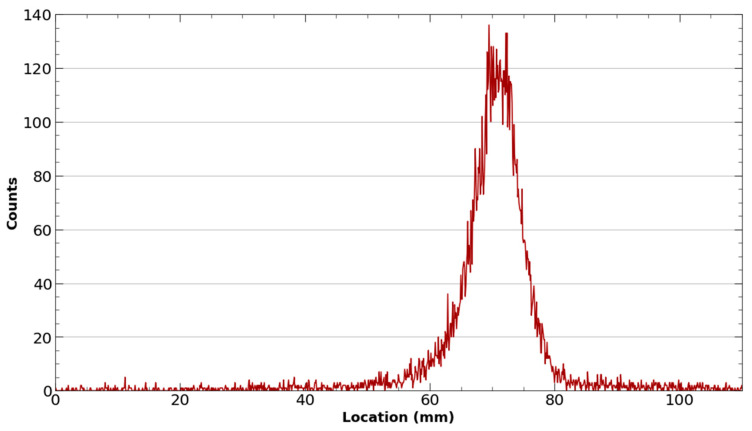
Representative iTLC of ^47^Sc-PSMA-617. The absence of a peak near the origin of the iTLC exhibits the successful radiolabeling of ^47^Sc-PSMA-617 with a yield of > 95%.

**Figure 11:**
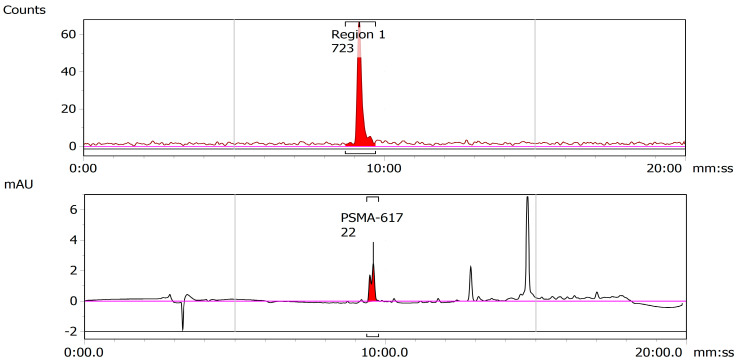
Representative HPLC chromatogram of ^47^Sc-PSMA-617. The proximity of the retention times of the UV trace of cold PSMA-617 (**bottom**) and the radioactive trace of the solutions of radiolabeled ^47^Sc-PSMA-617 and (**top**) indicate successful radiolabeling.

The dry ^47^ScCl_3_ residue, received from the Argonne National Laboratory, was reconstituted in 0.2 mL of 0.01 M HCl (trace analysis grade) in a 4 mL glass v-vial. The remaining radiolabeling process proceeded as described in 2.3.1 for ^43^Sc radiolabeling and yielded a specific activity of 8.07 MBq/nmol. A representative TLC and HPLC for ^47^SC are shown below in Figure 10 and Figure 11.

### 2.4. Stability of ^43/47^Sc-PSMA-617

**Figure 12:**
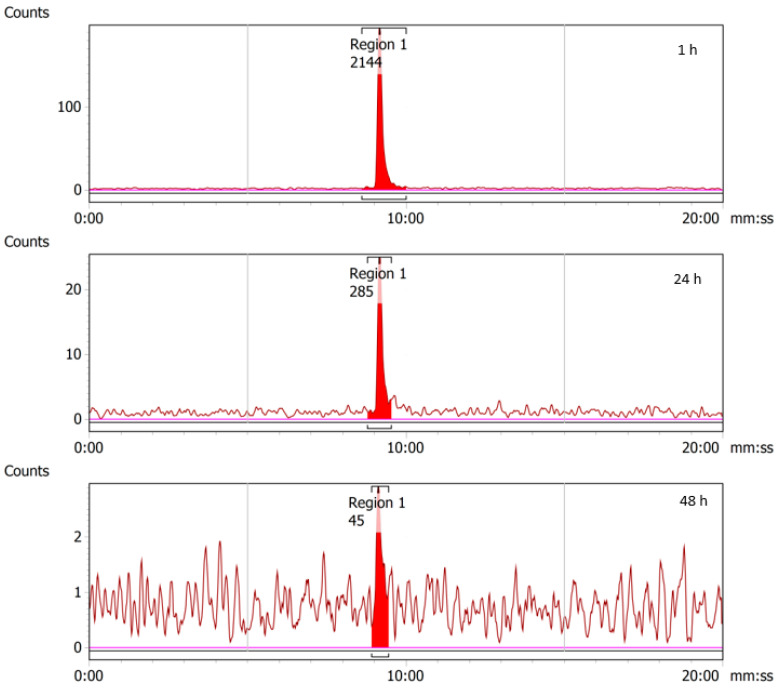
RadioHPLC chromatograms from 1, 24, and 48 h time-points for ^43^Sc-PSMA-617. As no additional peaks are observed beyond the main radiolabeled ^43^Sc-PSMA-617 peaks, it can be concluded that the compound is stable in PBS. Decreasing peak size is the result of radioactive decay.

**Figure 13:**
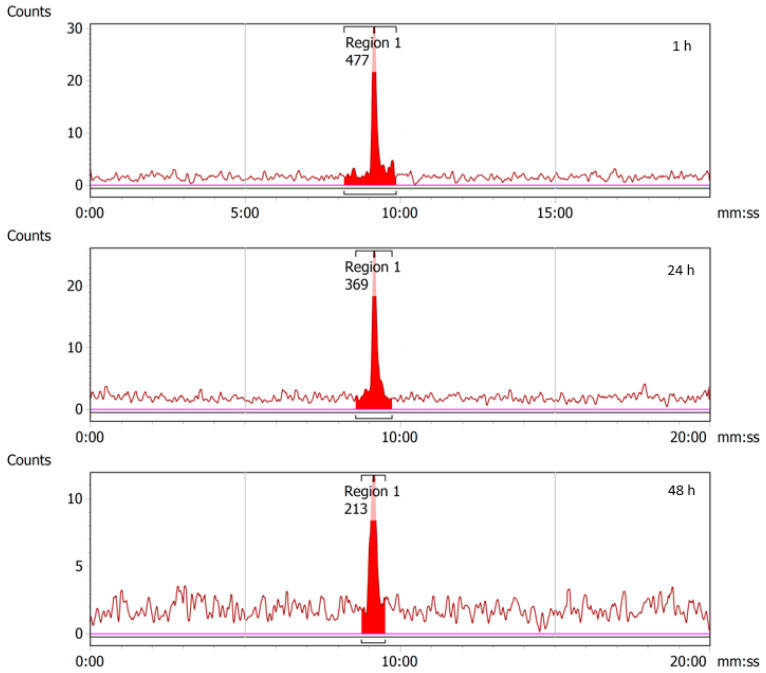
RadioHPLC chromatograms from 1, 24, and 48 h time-points for ^47^Sc-PSMA-617. As no additional peaks are observed beyond the main radiolabeled ^47^Sc-PSMA-617 peaks, it can be concluded that the compound is stable in PBS. Decreasing peak size is the result of radioactive decay.

The doses of radiolabeled ^43^Sc-PSMA-617 and ^47^Sc-PSMA-617 prepared in PBS were stability-checked over 24 and 48 h at ambient temperature, respectively, and were found to be stable over that period (Figure 12 and Figure 13).

### 2.5. In Vivo Animal Studies

#### 2.5.1. ^43^Sc-PSMA-617

**Figure 14:**
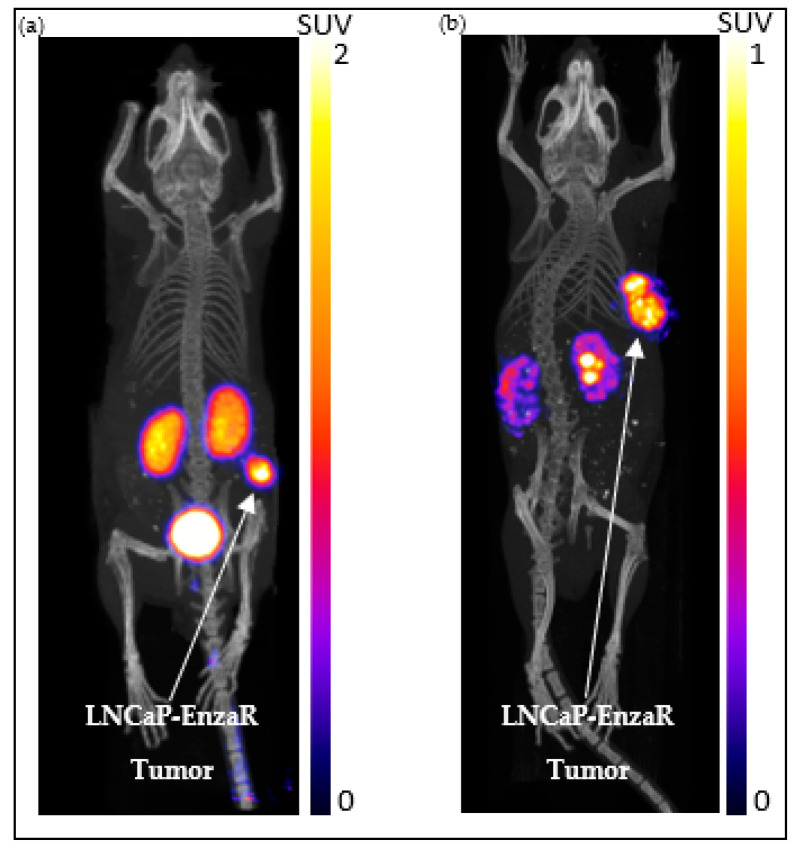
Representative images of the LNCaP-EnzaR animal model taken with ^43^Sc PET (**a**) and ^47^Sc SPECT (**b**) at 2 h post-injection. The arrows in each figure point to the xenografted LNCaP-ENZaR tumor.

PSMA-expressing LNCaP-EnzaR-Luc cells were implanted onto the right flank of an athymic nude mouse to produce a tumor xenograft. The mouse received a tail vein injection of 5.66 MBq of ^43^Sc-PSMA-617 and was allowed an uptake period of two hours. Following the uptake period, the animals underwent a 30-min PET acquisition, followed by a CT scan to obtain anatomical information. The resulting image is shown in Figure 14a, where the uptake is displayed in standardized uptake values (SUV).

The imaging revealed a significant uptake of ^43^Sc-PSMA-617 in the tumor, while the remaining uptake was limited to the kidneys and bladder. The standardized uptake value ratio (SUVR) of the tumor and muscle tissue corresponding to the contralateral side of the tumor (i.e., the left flank) was found to be 129.16. These findings show that ^43^Sc-PSMA-617 has a high specificity for PSMA expression and exhibits tumor uptake characteristic of other PSMA-617-based radiopharmaceuticals [28,29]. This suggests that ^43^Sc-PSMA-617 is viable in the pre-clinical imaging of prostate cancer.

#### 2.5.2. ^47^Sc-PSMA-617

An SCID mouse exhibiting a PSMA-expressing LNCaP-EnzaR-Luc tumor xenograft on the right flank was given 53.7 MBq of ^47^Sc-PSMA-617 through a tail vein injection. Two hours post-injection, animals were imaged with a 90-min SPECT scan followed by a CT scan for anatomical information; this is shown in Figure 14b. High uptake of ^47^Sc-PSMA-617 in the xenografted tumor was observed, with the remaining uptake being non-specific uptake in the renal system [10,30,31]. The SUVR of the tumor relative to contralateral muscle tissue was found to be 53.82. Hot spots in the kidney are the result of non-specific uptake in the renal calyces before excretion through the ureter. As uptake was limited to PSMA expression in the tumor xenograft and the renal system, these results have exhibited that ^47^Sc-PSMA-617 has a high degree of selectivity in PSMA-positive tumors, which is congruent with other PMSA-617-based radiopharmaceuticals [28,29,32]. As such, prostate cancer detection with ^47^Sc-PSMA-617 is feasible, and the biodistribution of radiotracer observed shows promise for future therapeutic evaluation.

## 3. Materials and Methods

### 3.1. Cyclotron-Based Production and Purification of ^43^Sc

#### 3.1.1. Beam-Stop as A Target Holder

The beam stop of a Cyclone 18/9 cyclotron (IBA, Louvain-la-Neuve, Brussels) was converted into a simple solid target holder to accommodate the use of ^42^CaO pellets for ^43^Sc production with a deuteron beam. The implementation of the solid target holder involved the precision machining of two aluminum disks, each having a diameter of 16 mm. The first disk was designed to incorporate a shallow well with a depth of 0.05 mm and diameter of 7.1 mm, providing sufficient space for the target material. The second disk was machined into a ring and possessed an outer diameter of 16 mm and an inner diameter of 7.1 mm. This disk had the primary function of securely attaching a thin carbon cover foil to the target holder. Four radially spaced, threaded M3 holes were machined into this backing disk with corresponding clear holes being machined into the ring. Two of these holes were employed for the purpose of joining the bracket and target holder, while the remaining two facilitated the manual retrieval of the target with a long M3 screw.

#### 3.1.2. Target Materials and Preparation

^42^CaO purchased from Isoflex USA had a ^42^Ca enrichment of 96.30% and was utilized for target synthesis in the first 10 productions indicated in Table 2. ^42^CaO containing an enrichment of 94.37% was purchased from Oak Ridge National Laboratory and was utilized in the remaining ^43^Sc productions. Targets were prepared by transferring the material into a pellet press (Parr Instrument Company, Moline, IL, USA, P/N 2810-2106-103101) and manually pressing each target. 

After the target was synthesized, two pieces of graphite foil (25 µ, Digi-Key, P/N EyG-S091203DP) were prepared by pre-coring two small holes through which the M3 screws passed when joining the target to the holder. The wooden handle (3.175 mm) of a simple swab was used to make the holes. The target was then placed between these two graphite foils on the backing plate of the target holder and then held in place by the retaining ring, which was secured by two M3 screws. Before the beam stop was inserted into the cyclotron port for irradiation, the carbon foil was trimmed, and diffusion pump oil was applied to the target holder backing to improve thermal transfer. 

#### 3.1.3. Target Irradiation

After mounting the beam stop with the target holder into the IBA Cyclone 18/9 medical cyclotron and bringing the system to vacuum, targets were irradiated with a 9 MeV deuteron beam at 1–8 µA for approximately 8 h. Targets were allowed to cool for 1 h prior to retrieval to minimize the dose from short-lived nuclides to personnel involved in target retrieval. 

Once the short-lived nuclides had died out, the target gate valve was closed, and the target was vented. Immediately after venting, the beam stop was removed and two long M3 screws were inserted into the empty screw holes of the target holder for manual retrieval of the target.

### 3.2. Accelerator Production and Purification of ^47^Sc

#### 3.2.1. Argonne National Laboratory LEAF

Argonne houses a variable-energy with variable-power L-band (1300 Hz) electron linear accelerator with a maximum energy of 53 MeV with an average beam power of up to 25 KW at 30 MeV. It can produce steady-state pulses that are continuously variable from 200 ns to 5.5 µs. The maximum repetition rate is 210 Hz. The peak current for the microsecond pulse can achieve 2 amps. The effective beam energy range is from 20–45 MeV. 

Bremsstrahlung photons are generated by bombarding a high “Z” converter with the incident electron beam. A Ta converter was employed for these experiments. Briefly, six Ta plates (each 0.5 mm thick) spaced ~1.0 mm apart were enclosed in an aluminum carrier with cooling channels. The convertor was cooled with 0.16 L/s flow of water directly over the plates.

#### 3.2.2. Irradiation

In a representative case, three pressed metal powder pellets were prepared by pressing natural Ti metal powder into 1 g 12.7 mm diameter pellets with a hydraulic pellet press (1 ton for 5 min). The three 1 g pellets were stacked and loaded into a water-cooled aluminum target holder. The target holder was placed behind and proximal to a converter and this system was irradiated for ~10–12 h with beam energies of 40 MeV and ~2.5–3 kW beam power using a beam spot of 6 × 6 mm. The temperature of the target holder was monitored and kept below 200 °C throughout the irradiations.

#### 3.2.3. Ti Metal Target Processing

The targets were allowed to decay for ~12 h prior to retrieval. The Ti pellets (~3 g) were dissolved in concentrated HCl (60 mL). The solution was refluxed with a condenser for 1 h, whereupon the solution became dark purple/blue. The solution was allowed to cool to room temperature, then transferred to a 100 mL graduated flask, and diluted to the mark with water. This ^47^Sc stock solution was sampled for gamma-ray analysis and inductively coupled plasma mass spectroscopy utilizing an HPGe detector (Ortec, Oak Ridge, TN, USA) and NexION 2000 ICP Mass Spectrometer (PerkinElmer, Waltham, MA, USA), respectively. Gamma peak analysis was performed using GammaVision version 7.02.01 software. ICP-MS analysis was performed with Syngistix Version 2.5.

The ^47^Sc was isolated from the Ti target material using a gravity-fed DGA resin column (0.75 g). The DGA resin (Eichrom, Lisle, IL, USA) was primed with 6 M HCl and then loaded with the ^47^Sc stock solution. The column was washed twice with HCl (3 M, 20 mL each), once with HNO_3_ (3 M, 20 mL), and a final HCl (1 M, 5 mL) wash. The ^47^Sc was eluted with warm HCl (0.1 M, 15 mL). The product fractions were combined and evaporated to dryness. The dry residue, containing ^47^Sc, ^46^Sc, and ^48^Sc, was packaged and shipped to the University of Chicago for testing.

#### 3.2.4. DOTA Titrations

The apparent molar activity (AMA) of the isolated ^47^Sc was determined by complexation with varying amounts of DOTA. Five vessels were labeled as D0, D1, D1, D3, and D4. Each tube was loaded with ammonium acetate buffer (1.0 M, pH 5.5, 100 µL). DOTA was added to each tube from a standard DOTA stock solution to provide 16.3 µg, 1.63 µg, 0.163 µg, 0.0489 µg, and 0.00163 µg of DOTA to the D0, D1, D2, D3, and D4 tubes, respectively. These solutions were spiked with ^47^Sc (3.7–7.4 MBq) and then incubated at 90 °C for 30 min. Each tube was spotted on two separate silica get TLC plates, which were developed using an EDTA (50 mM) or NH_4_OH (10% *w*/*v*)/methanol solution (1:1).

### 3.3. Radiolabeling of PSMA-617 with ^43^Sc and ^47^Sc

^43^Sc/^47^ScCl_3_ was reconstituted in 0.01 M HCl and pH was adjusted to 4.0 by 1 M ammonium acetate buffer to a final buffer concentration of 0.5 M. DOTA-PSMA-617 was added and the reaction was mixed for 45 min at 95 °C on a thermomixer at 450 rpm. The resulting ^43^Sc/^47^Sc labeled ligands were then trapped on a preconditioned C18 SPE cartridge (Waters, Milford, MA, USA). The column was washed with water. The purified product was eluted with ethanol, dried and reconstituted in PBS for cellular and animal studies.

### 3.4. Quality Control of ^43^Sc and ^47^Sc Labeled Radioligands

Characterization of the synthesized probes was done by iTLC-SG (glass microfiber chromatography paper impregnated with silica gel, Agilent Technologies, Santa Clara, CA, USA) and HPLC (Infinity 1260 series, Agilent Technologies, Santa Clara, CA, USA) equipped with a Flow-Ram radioHPLC detector (LabLogic, Sheffield, UK). Typically, a 50% MeOH solution in 1 M ammonium formate is used to develop the iTLC papers and then evaluated with a Scan-Ram radioTLC scanner (LabLogic, Sheffield, UK).

### 3.5. Cell Culture for Xenograft

PSMA-expressing parental LNCaP or the luciferase-expressing enzalutamide-resistant LNCaP-EnzaR-Luc cells were used [33]. All human prostate cancer cells were authenticated at the University of Arizona and tested regularly for mycoplasma using the MycoAlert Plus kit (Lonza, Basel, Switzerland). Cells were maintained in RPMI1640 medium supplemented with 10% fetal bovine serum under humidified conditions at 37 °C with 5% CO_2_. In addition, the maintenance medium for the LNCaP-EnzaR cells contains 10 µM enzalutamide (Astellas, Tokyo, Japan) and 300 µg/mL hygromycin B (Invitrogen, Waltham, MA, USA).

### 3.6. Animal Preparation

Animal experiments were performed in the context of an IACUC-approved animal protocol (P.I. Szmulewitz). As previously described, 2 × 10^6^ luciferase-expressing LNCaP-EnzaR were resuspended in 35 µL Hanks Balanced Salt Solution (HBSS) (Corning, Corning, NY, USA) and mixed with 115 µL Matrigel (Corning, Corning, NY, USA) and inoculated subcutaneously into an immunocompromised 8–10 week-old SCID or athymic nude mouse (Harlan, Indianapolis, IN, USA) [33]. Animals were monitored to determine when the xenograft was established, determined by the tumor being palpable and visualized using bioluminescence imaging prior to ^43^Sc/^47^Sc-PSMA-617 imaging.

### 3.7. In Vivo Imaging of Tumor-Bearing Mouse Models with ^43^Sc/^47^Sc-PSMA-617

#### 3.7.1. Static microPET/CT Imaging with ^43^Sc-PSMA-617

The animal model was injected with approximately 5.66 MBq of ^43^Sc-PSMA-617 in 100 µL of PBS. After a 2 h awake uptake period, micro CT and microPET scans were acquired on the X-Cube and β-Cube microCT and microPET systems, respectively (Molecubes, Gent, Belgium). A general purpose protocol was used for microCT and a 30 min acquisition time for microPET scans. MicroPET images were reconstructed using an OSEM reconstruction algorithm with an isotropic voxel size of 400 μm. CT images were reconstructed with a 200 μm isotropic voxel size and used for anatomic co-registration and scatter correction. The animal was maintained under 1–2% isoflurane anesthesia in 100% oxygen during imaging. Respiratory rate and body temperature were constantly monitored and maintained using Molecubes onboard monitoring and a Small Animal Instruments (SAII Inc, Stoney Brook, NY, USA) setup. Images were coregistered and post-processed using VivoQuant software (InviCRO, Boston, MA, USA, https://www.vivoquant.com/, accessed on 19 July 2023). Utilizing the CT scans, regions of interest were drawn around tumors and the muscle tissue corresponding to the contralateral side of the tumor. Data are shown in standard uptake volume.

#### 3.7.2. Static microSPECT/CT Imaging with ^47^Sc-PSMA-617

An animal model was injected with 53.9 MBq of ^47^Sc-PSMA617 in 100 µL of isotonic saline solution. After 2 h incubation, animals were anesthetized with 1–2% isoflurane, placed in the γ-Cube microSPECT instrument (Molecubes, Gent, Belgium) and scanned for 90 min, followed by microCT scan for anatomic reference.

## 4. Conclusions

The production of ^43^Sc via the ^42^Ca(d,n)^43^Sc reaction was found to be feasible using a deuteron-capable medical cyclotron. Radioactivity was readily produced at a level capable of supporting pre-clinical studies with the addition of an easily fabricated beam stop modification. Similarly, ^47^Sc was shown to be readily producible with the ^Nat^Ti(γ,p)^47^Sc reaction at a linear accelerator with off-site production proving to be no obstacle for a pre-clinical study. ^43^Sc and ^47^Sc produced through these methods were successfully used for labeling PSMA-617 at specific activities appropriate for preliminary in vivo studies. Clear detection of LNCaP-r tumors was possible with both ^43^Sc-PSMA-617 PET and ^47^Sc-PSMA-617 SPECT imaging.

Together, these preliminary findings exhibit ^43^Sc/^47^Sc as a promising theranostic pair. Through the experimentally demonstrated pipeline defined by this publication, ^43^Sc/^47^Sc is easily adaptable to the pre-clinical space. As such, this theranostic pipeline will be utilized to vastly expand upon the dataset presented in this publication through the full-scale investigation of ^43^Sc/^47^Sc-PSMA-617 with subsequent expansion to the utilization of scandium theranostics with other DOTA based tracers.

This paper also illustrates the initial progress of the UChicago/Argonne Joint Radioisotope Initiative’s (JRI) efforts in building a complete pipeline for rapid development of novel theranostics.

## Figures and Tables

**Table 1:** Isotopic breakdown of the ^42^Ca from Oak Ridge National Laboratory and Isoflex USA utilized in this study. All potential reaction cross-sections from deuteron bombardment are presented.

*Calcium Isotope*	Abundance (ORNL)	Abundance (Isoflex USA)	Reaction	Product Half-Life	IAEA ENDF Cross-Section at 9 MeV (×10^−27^ cm^−1^)
^40^Ca	5.01%	3.03%	^40^Ca(d,p)^41^Ca ^40^Ca(d,n)^41^Sc ^40^Ca(d,α)^38^K	9.94 × 10^4^ y 596.3 ms 924.4 ms	369.00 41.68 30.70
^42^Ca	94.37%	96.30(±0.30)%	^42^Ca(d,p)^43^Ca ^42^Ca(d,n)^43^Sc ^42^Ca(d,α)^40^K	Stable 3.89 h 1.25 × 10^9^ y	266.74 104.73 60.08
^43^Ca	0.06%	0.10%	^43^Ca(d,p)^44^Ca ^43^Ca(d,n)^44^Sc ^43^Ca(d,2n)^43^Sc ^43^Ca(d,α)^41^K	Stable 3.97 h 3.89 h Stable	105.31 128.42 228.04 65.85
^44^Ca	0.55%	0.55%	^44^Ca(d,p)^45^Ca ^44^Ca(d,n)^45^Sc ^44^Ca(d,2n)^44^Sc ^44^Ca(d,α)^42^K	162.61 d Stable 3.97 h 12.36 h	84.75 262.34 264.24 24.45
^46^Ca	0.001%	<0.01%	^46^Ca(d,p)^47^Ca ^46^Ca(d,n)^47^Sc ^46^Ca(d,2n)^46^Sc ^46^Ca(d,α)^44^K	4.54 d 3.35 d 83.79 d 22.13 m	215.50 155.62 620.39 7.11
^48^Ca	0.01%	0.02%	^48^Ca(d,p)^49^Ca ^48^Ca(d,n)^49^Sc ^48^Ca(d,2n)^48^Sc ^48^Ca(d,α)^46^K	8.72 m 57.18 m 43.67 h 105 s	40.09 93.64 1116.59 2.12

**Table 2:** Representative ^43^Sc experimental yields as a function of target mass, beam current, and irradiation time. Grey shading indicates use of the 96.30% enriched ^42^CaO from Isoflex USA. No shading indicates the use of 94.37% enriched material from ORNL.

Target Mass (mg)	Beam Current (μA)	Irradiation Time (h)	EOB Yield (MBq)
16.4	1	8	163.54
9.3	2	10	185.37
5.8	3	10	163.54
4.6	3	7.5	159.10
18.2	5	7.5	663.04
21.9	4	8.0	839.90
16.4	4	8.0	685.24
19.3	4	8.0	715.95
19.0	4	8.0	694.12
7.0	4	8.0	247.53
13.3	4	10.0	654.53
16.5	4	9.0	433.27
13.4	4	8.0	408.85
12.3	4.2	8.0	515.04
22.4	6	7.72	1229.14
14.5	6	8	1083.36
13.4	7.75	8.12	1162.91
17.0	7.25	8	1224.70
13.4	8	8	1569.54
17.0	8	8	1627.26
17.2	8	8	1326.45
14.5	8	8	1087.80
17.6	8	8	1211.88

## Data Availability

The data presented in this study are available on request from the corresponding authors. The data are not publicly accessible because they are not available in a format that is sufficiently accessible or reusable by other researchers.

## Sample Availability

Not available.
